# Dietary Mineral Intake and Vascular Health in Patients with Long COVID-19: The BioICOPER Study

**DOI:** 10.3390/nu18132140

**Published:** 2026-07-02

**Authors:** Alicia Navarro-Cáceres, Elena Navarro-Matías, Silvia Arroyo-Romero, Nuria Suárez-Moreno, Andrea Domínguez-Martín, Cristina Lugones-Sanchez, Susana Gonzalez-Sanchez, Manuel A. Gómez-Marcos, Marta Gómez-Sánchez, Leticia Gómez-Sánchez, BioICOPER Investigators Group

**Affiliations:** 1 Unidad de Investigación en Atención Primaria de Salamanca (APISAL), Gerencia de Atención Primaria de Salamanca, Gerencia Regional de Salud de Castilla y León (SACyL), Avenida de Portugal 83, 37005 Salamanca, Spain; alicia.nav@usal.es (A.N.-C.); enavarro@saludcastillayleon.es (E.N.-M.); silvia_ar@usal.es (S.A.-R.); nuria.suarez@usal.es (N.S.-M.); andreadm@usal.es (A.D.-M.); cristinals@usal.es (C.L.-S.); gongar04@gmail.com (S.G.-S.); 2 Instituto de Investigación Biomédica de Salamanca (IBSAL), Paseo de San Vicente, 58-182, 37007 Salamanca, Spain; 3 Departamento de Medicina, Universidad de Salamanca, Calle Alfonso X el Sabio s/n, 37007 Salamanca, Spain; 4 Red de Investigación en Cronicidad, Atención Primaria y Promoción de la Salud (RICAPPS), Avenida de Portugal 83, 37005 Salamanca, Spain; 5 Servicio de Hospitalización a Domicilio, Hospital Universitario Marqués de Valdecilla, 39008 Santander, Spain; martagmzsnchz@gmail.com; 6 Servicio de Urgencias, Hospital Universitario La Paz, Paseo de la Castellana 261, 28046 Madrid, Spain; leticiagmzsnchz@gmail.com

**Keywords:** long COVID-19, arterial stiffness, vascular health, dietary mineral intake, vascular aging

## Abstract

**Background/Objectives:** Long COVID-19 (LC) has been associated with persistent inflammation and impaired vascular health. Dietary minerals are involved in oxidative stress, endothelial homeostasis, and arterial stiffness; however, their relationship with vascular health in LC remains poorly explored. This study aimed to examine the association between energy-adjusted dietary mineral intake and markers of vascular stiffness and vascular aging in adults with LC, while exploring potential sex-specific patterns. **Methods:** A total of 304 adults with LC from the BioICOPER study were included. Dietary mineral intake was assessed using a validated 7-day dietary record from the EVIDENT tool and expressed as mineral density per 1000 kcal for the regression analyses. Vascular assessment included carotid intima-media thickness (cIMT), carotid-femoral pulse wave velocity (cfPWV), brachial-ankle pulse wave velocity (baPWV), and the vascular aging index (VAI). Hierarchical multivariable linear regression models, false discovery rate (FDR) correction, restricted cubic spline analyses, sensitivity analyses excluding supplement users, and formal sex × mineral interaction tests were performed. **Results:** In descriptive adequacy analyses, adequate iron intake was associated with lower baPWV. In energy-adjusted linear regression models, no mineral-outcome association remained statistically significant after FDR correction. In the fully adjusted sensitivity model, zinc density showed a nominal positive association with cfPWV, but this association did not survive FDR correction. Restricted cubic spline analyses suggested possible non-linear associations of magnesium and potassium density with cfPWV and VAI. Formal interaction analyses did not provide robust evidence of sex-related effect modification. **Conclusions:** After energy adjustment and correction for multiple testing, the evidence for independent linear associations between dietary mineral density and vascular outcomes in adults with LC was limited. These exploratory findings suggest that mineral intake, dietary sources, and non-linear patterns deserve further evaluation in prospective studies and nutritional intervention trials.

## 1. Introduction

Long COVID-19 (LC) represents one of the most complex public health challenges worldwide [1,2,3]. Beyond its heterogeneous clinical manifestations, increasing evidence indicates that LC is characterized by a state of chronic low-grade inflammation and sustained endothelial dysfunction [4,5,6]. The CARTESIAN study has shown that persistent viral-related inflammation may promote accelerated vascular aging, characterized by structural elastin degradation and abnormal collagen deposition within the extracellular matrix of arterial walls [7]. Consequently, endothelial impairment may contribute to increased arterial stiffness, which can persist for months or even years after the initial infection [4,7]. In addition, the vascular consequences of LC may differ according to sex, with women with LC showing poorer arterial elasticity in some studies [8,9]. Accordingly, the assessment of arterial stiffness, an independent predictor of cardiovascular morbidity and mortality, has become a relevant clinical and research tool for monitoring subclinical cardiovascular risk in individuals with LC [10,11,12]. A comprehensive evaluation of vascular health requires tools capable of assessing different arterial segments and properties of the vascular wall. Carotid–femoral pulse wave velocity (cfPWV) remains the gold-standard measure of central arterial stiffness [13,14,15], whereas brachial–ankle pulse wave velocity (baPWV) provides information on peripheral arterial stiffness [10,16,17]. In turn, the vascular aging index (VAI) integrates carotid intima–media thickness (cIMT) and cfPWV, thereby capturing both structural and functional components of vascular aging [18]. Taken together, these vascular markers may reflect the dynamic nature of persistent endothelial injury, driven by chronic oxidative stress and impaired vascular repair capacity in patients with LC [19,20].

Dietary composition plays a key role in arterial stiffness and vascular health [21,22]. In the context of the vascular vulnerability observed in individuals with LC, nutritional status, and particularly dietary mineral intake, may emerge as a potentially relevant modifiable factor [23]. Several minerals are involved in endothelial protection, oxidative balance, inflammatory regulation, vascular tone, and arterial wall remodeling. Magnesium (Mg), for instance, acts as a natural calcium antagonist and as a cofactor in nitric oxide synthesis, thereby helping to prevent vascular calcification and maintain arterial distensibility [24]. Magnesium deficiency, whether due to low intake or metabolic depletion, may promote oxidative stress, endothelial dysfunction, and inflammation [25,26], and this cardiovascular risk may be amplified when accompanied by high copper levels or low zinc availability [27]. In the context of COVID-19, infection-related alterations in magnesium homeostasis may contribute to vascular hypertonia, elastic fiber degradation, and fibrosis. Although short-term supplementation with magnesium citrate has not been shown to reverse arterial stiffness [28], lower dietary magnesium intake has been associated with greater clinical severity and neuropsychiatric sequelae [29,30]. Moreover, low serum magnesium levels at the onset of infection have been associated with a higher risk of developing persistent post-COVID-19 symptoms [31]. Selenium (Se) is involved in antioxidant defense, inflammatory regulation, and immune response. Selenium deficiency may promote oxidative stress, endothelial dysfunction, low-grade inflammation, and reduced nitric oxide bioavailability, all of which are closely linked to arterial stiffness [32,33]. In both high-cardiovascular-risk populations and the general population, inadequate selenium intake or altered selenium status has been associated with vascular stiffness, suggesting that both deficiency and excess may be unfavorable for vascular health [32,34]. In COVID-19, low selenium concentrations have been associated with worse clinical outcomes and higher mortality, while systematic reviews indicate that patients with COVID-19 commonly show lower selenium levels than healthy controls, with deficiency being related to greater disease severity [35,36,37,38]. Iodine deficiency has been associated with higher all-cause mortality, while thyroid hormone alterations have been linked to arterial stiffness and vascular dysfunction [39,40]. Furthermore, COVID-19 may induce thyroid dysfunction during the acute phase and in individuals with LC, including thyroiditis and alterations in thyroid hormone levels [41]. Calcium (Ca) and phosphorus (P) are also essential for bone, energy, and vascular homeostasis; however, disturbances in calcium–phosphorus balance may promote endothelial dysfunction, vascular calcification, and increased cardiovascular risk, particularly in the presence of hyperphosphatemia or persistent metabolic alterations [42,43]. Recent reviews suggest that phosphate may contribute to cardiovascular disease through vascular calcification pathways, although contemporary epidemiological data do not support a direct association between higher dietary calcium or phosphorus intake per se and greater vascular or valvular calcification in the general population [44]. In COVID-19, disturbances in calcium, phosphorus, and magnesium metabolism during and after infection have been associated with poorer clinical outcomes [45]. Sodium (Na) and potassium (K) intake are central to extracellular volume regulation, blood pressure control, endothelial function, and vascular tone. High sodium intake and low potassium intake have been associated with higher blood pressure and poorer vascular function. In addition, evidence on arterial stiffness suggests that excess sodium intake, particularly when accompanied by low potassium intake, may contribute to increased pulse wave velocity, whereas sodium restriction can reduce arterial stiffness independently of blood pressure lowering [46]. Conversely, greater potassium availability or supplementation has been associated with improved endothelial function, which may help modulate the vascular impact of a sodium-rich diet [47]. In COVID-19, electrolyte disturbances, including abnormalities in sodium and potassium, have been associated with worse clinical evolution [48]. Zinc (Zn) is an essential trace element with antioxidant, anti-inflammatory, and immunomodulatory properties, and is involved in endothelial homeostasis and protection against oxidative stress. Insufficient zinc intake has been related to cardiovascular risk in a non-linear manner, suggesting that intake should be adequate but not excessive [49]. In patients with high vascular risk, zinc deficiency has also been independently associated with greater arterial stiffness [50]. In the context of COVID-19, zinc deficiency has been associated with poorer clinical outcomes and with a higher risk of hospitalization and mortality during the post-acute phase [51]. Finally, iron (Fe) is essential for oxygen transport and mitochondrial function, but its homeostasis requires tight regulation, as both deficiency and functional iron excess may contribute to inflammation, oxidative stress, and vascular damage. Regarding arterial stiffness, higher ferritin levels have been associated with greater pulse wave velocity in patients with hypertension and with higher baPWV in healthy adults [52]. Moreover, evidence supports a causal relationship between higher iron status, including serum iron, ferritin, and transferrin saturation, and increased arterial stiffness [53]. In COVID-19, dysregulation of iron metabolism and anemia are frequent during follow-up and have been related to persistent symptoms and poorer functional recovery [54].

Overall, previous evidence supports the relevance of minerals in vascular health, while also highlighting the lack of specific data in individuals diagnosed with LC. Therefore, it is necessary to examine the association between dietary mineral intake and vascular health in this population using validated dietary assessment instruments, such as those developed within the EVIDENT study [55], together with a comprehensive evaluation of the arterial tree.

Based on the hypothesis that dietary intake of essential minerals may be related to functional and structural vascular alterations in individuals with LC, this study was designed with two main objectives. First, we aimed to evaluate the association between energy-adjusted dietary mineral intake, expressed as mineral density per 1000 kcal, and target indices of arterial stiffness and vascular aging, including cfPWV, baPWV, and VAI, in adults diagnosed with LC from the BioICOPER project. Second, we sought to explore whether these nutritional-vascular relationships differed according to biological sex using formal interaction testing, while interpreting sex-stratified analyses as exploratory.

## 2. Materials and Methods

### 2.1. Study Design and Participants

The BioICOPER cohort included 304 adults with long COVID-19. Descriptive analyses were performed in the overall available sample. Regression analyses were conducted using available-case samples according to the availability of dietary, vascular, and covariate data. The study was conducted at the Primary Care Research Unit of Salamanca (APISAL). The present analysis forms part of the BioICOPER project, registered in 18 April 2023 at ClinicalTrials.gov (Identifier: NCT05819840). The study protocol has been previously published [56].

**Figure 1:**
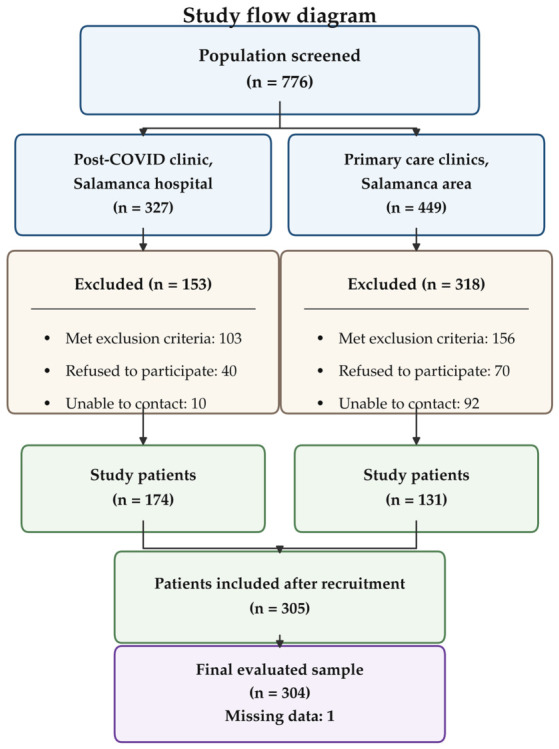
Flowchart of participant selection, including inclusion and exclusion criteria, in the BioICOPER study.

Participants were recruited by consecutive sampling. The inclusion criteria were as follows: diagnosis of LC according to the World Health Organization (WHO) definition [57], namely a history of probable or confirmed SARS-CoV-2 infection and the presence of symptoms lasting for at least 2 months that could not be explained by an alternative diagnosis; and provision of written informed consent. The exclusion criteria were terminal illness, inability to attend the primary care center for assessment, a history of cardiovascular disease, including ischemic heart disease or cerebrovascular disease, or an estimated glomerular filtration rate below 30 mL/min/1.73 m^2^. The participant flow chart is shown in Figure 1.

#### Sample Size Calculation

Sample size and detectable effect size were estimated using GRANMO software v.5 (https://www.datarus.eu/ca/aplications/granmo/, accessed on 18 April 2026). For the multiple linear regression analyses, considering a total sample size of 304 participants, an alpha risk of 0.05, a beta risk below 0.20, the number of predictors included in the fully adjusted model, and, conservatively, the lowest adjusted coefficient of determination observed in the final models —cfPWV: adjusted R^2^ = 0.347—, the available sample size was considered sufficient to assess the association between dietary magnesium intake and the main vascular parameters analyzed. In addition, assuming a standard deviation of 82 mg/day for magnesium intake, the sample of 304 participants allowed the mean dietary magnesium intake to be estimated with an approximate precision of ±9.2 mg/day for a 95% confidence interval.

The present study was conducted in accordance with the Strengthening the Reporting of Observational Studies in Epidemiology (STROBE) guidelines [58]. The completed STROBE checklist is provided in Supplementary Table S1.

### 2.2. Variables and Measurement Instruments

To reduce information bias, all analyzed variables and complementary assessments were collected by four healthcare professionals who had been trained before the start of the study and followed a standardized protocol. Data were recorded using Research Electronic Data Capture (REDCap), hosted by the Biomedical Research Institute of Salamanca (IBSAL), for data collection and management. Quality control was performed by an independent researcher [56].

### 2.3. Dietary Intake Assessment

Dietary mineral intake was assessed using a food record collected through the EVIDENT mobile application [55]. This application was developed and validated by the Primary Care Research Group of Castilla y León, affiliated with REDIAPP, and is registered under intellectual property number 00/2014/2207. Participants recorded all foods and beverages consumed over seven consecutive days, including portion sizes. Foods were classified into predefined groups within the EVIDENT tool. Daily mineral intake was estimated using Spanish food composition tables and included iron (Fe), magnesium (Mg), selenium (Se), iodine (I), calcium (Ca), phosphorus (P), sodium (Na), potassium (K), and zinc (Zn) [55]. To account for the influence of total energy intake, energy-adjusted mineral intake variables were additionally calculated and expressed as mineral intake per 1000 kcal. These energy-adjusted variables were used in the regression analyses to better assess the association between dietary mineral density and vascular parameters and to minimize the possibility that observed associations reflected overall dietary quantity rather than mineral-specific intake. When fortified foods were recorded by participants and were available as such in the food composition database used by the EVIDENT tool, their mineral content was incorporated as part of the corresponding food item. However, the dietary assessment tool did not allow separate quantification of minerals derived specifically from food fortification, nor did it distinguish naturally occurring minerals from minerals added during food processing or enrichment. Therefore, mineral intake estimates should be interpreted as total dietary intake from recorded foods according to the available food composition data, without separate attribution to fortification. Dietary supplement intake was not quantified by the dietary record; available self-reported supplement use was therefore considered separately in sensitivity analyses. Adequate and inadequate/risk intake groups were defined using mineral-specific reference values derived from the European Food Safety Authority (EFSA) Dietary Reference Values [59] for nutrients and the Spanish Society of Community Nutrition (SENC) nutritional objectives/recommended intakes for the Spanish population [60]. Sex-specific cut-off points were applied when available. Participants whose intake met or exceeded the corresponding reference value were classified as having adequate intake, whereas those below the cut-off point were classified as having inadequate or at-risk intake. Except in the case of Na, which is considered adequate if Na intake is <2000 mg per day. The cut-off points used for each mineral are presented in Supplementary Table S2.

### 2.4. Assessment of Vascular Structure, Function, and Aging

All vascular measurements were performed after 10 min of rest in the supine position, in a temperature-controlled room.

#### 2.4.1. Carotid Intima–Media Thickness Measurement

Carotid intima–media thickness (cIMT) was measured by two investigators with demonstrated reliability. Interobserver and intraobserver intraclass correlation coefficients (ICCs), calculated in 20 participants before the start of the study, were 0.974 and 0.897, respectively. Measurements were performed using a Sonosite Micromax^®^ ultrasound system (Sonosite Inc., Bothell, WA, USA), equipped with a high-resolution 5–10 MHz multifrequency linear transducer and Sonocal software 1.0 (Washington, DC, USA), which enables automated cIMT measurement. cIMT assessments were performed according to the protocol previously published by our research group [61].

#### 2.4.2. Arterial Stiffness Measurements

Arterial stiffness was assessed by measuring carotid–femoral pulse wave velocity (cfPWV) and brachial–ankle pulse wave velocity (baPWV).

Central arterial stiffness was assessed using cfPWV, measured with the SphygmoCor system (AtCor Medical Pty Ltd., West Ryde, Australia). Pulse waves were recorded at the carotid and femoral arteries. Transit time was estimated in relation to the R wave of the electrocardiogram. Distances were measured with a measuring tape from the suprasternal notch to the carotid and femoral recording sites. cfPWV was calculated according to established measurement guidelines [13,14,15].

baPWV was measured using the VaSera VS-2000 device (Fukuda Denshi Co., Ltd., Tokyo, Japan). Electrodes were placed on both arms and ankles, and measurements were obtained with the participant in the supine position, remaining silent and still. Measurements were considered valid after at least three consecutive stable cardiac cycles. According to the manufacturer’s instructions, baPWV was calculated using the following equation:

baPWV = (0.5934 × height (cm) + 14.4724)/t_ba

baPWV was derived from the ratio between the estimated arterial path length, based on body height, and the pulse transit time between the brachial and ankle sites, reflecting arterial stiffness [10,16,17].

#### 2.4.3. Vascular Aging Index Measurement

The vascular aging index (VAI) was calculated as previously described by Wadström et al. [18]. The index was derived by standardizing cIMT and cfPWV into z-scores and summing both components, according to the following formula:

VAI=(cIMT−mean cIMT)/SD cIMT+(cfPWV−mean cfPWV)/SD cfPWV

### 2.5. Sociodemographic Variables, Lifestyle Factors, and Laboratory Analyses

Sociodemographic and lifestyle variables were collected using standardized questionnaires. Age and sex were recorded, together with lifestyle factors such as tobacco and alcohol consumption, which were assessed using questionnaires adapted from the WHO MONICA study [62]. Adherence to the Mediterranean diet was evaluated using the 14-item Mediterranean Diet Adherence Screener (MEDAS) [63]. Physical activity was assessed using the short version of the International Physical Activity Questionnaire (IPAQ-SF) [64], with results expressed as metabolic equivalent task minutes per week (MET-min/week). Health-related quality of life was evaluated using the 36-item Short Form Health Survey (SF-36) [65].

Fasting blood samples were obtained to determine lipid profile and plasma glucose levels. Blood pressure and heart rate were measured using a validated automated sphygmomanometer (OMRON M10-IT; Omron Healthcare Co., Ltd., Kyoto, Japan), following the recommendations of the European Society of Hypertension [14].

### 2.6. Statistical Analysis

Statistical analyses were performed using IBM SPSS Statistics version 30.0 (IBM Corp., Armonk, NY, USA) and R version 4.4.2 (R Foundation for Statistical Computing, Vienna, Austria). For descriptive analyses, quantitative variables were expressed as mean ± standard deviation (SD), and comparisons between men and women were performed using Student’s *t*-test for independent samples. Qualitative variables were expressed as frequencies and percentages, and comparisons between sexes were performed using the chi-square test. Analysis of covariance (ANCOVA) was used to compare adjusted estimated marginal means of vascular function and vascular aging parameters between participants with adequate and inadequate mineral intake. Results were expressed as estimated marginal means and 95% confidence intervals (95% CIs). These adequacy analyses were considered descriptive because mineral adequacy was defined according to established intake cut-off points. Multiple linear regression models were used to evaluate the association between dietary mineral density and vascular function and vascular aging parameters. cfPWV, baPWV, and VAI were included as dependent variables. Energy-adjusted mineral intake variables, expressed as mineral intake per 1000 kcal, were entered individually as independent variables. The minerals analyzed were calcium (Ca), iron (Fe), iodine (I), phosphorus (P), potassium (K), magnesium (Mg), selenium (Se), sodium (Na), and zinc (Zn). Regression analyses were performed using a hierarchical modeling strategy. Model 1 was adjusted for age, sex (0 = men; 1 = women), smoking status (0 = non-smoker; 1 = smoker), physical activity expressed as MET-min/week, and alcohol consumption in grams. Energy adjustment was incorporated through the use of mineral density variables expressed per 1000 kcal. Model 2, considered the fully adjusted sensitivity model, further included Mediterranean diet score, SF-36 score, hypertension, type 2 diabetes mellitus, dyslipidemia, and abdominal obesity. Because cardiometabolic variables such as hypertension, diabetes, dyslipidemia, and obesity may lie on the causal pathway between dietary intake and vascular stiffness, Model 2 was interpreted cautiously as a conservative sensitivity analysis. Before performing the analyses, the assumptions of the linear regression models were checked. Multicollinearity was assessed using the variance inflation factor (VIF), with all values being <2.0. Results were expressed as unstandardized regression coefficients (β) with their corresponding 95% CIs. Correction for multiple comparisons was performed using the Benjamini–Hochberg procedure to control the false discovery rate across the pre-specified mineral-outcome contrast family. This family comprised nine minerals, three vascular outcomes, and two adjustment models, yielding 54 contrasts. Statistical significance for the association analyses between mineral density and vascular outcomes was determined using FDR-adjusted q-values, with q < 0.05 considered statistically significant. Additional sensitivity summaries examined FDR correction within restricted contrast families. To address the possibility of non-linear relationships, additional analyses were performed using restricted cubic splines with mineral density variables as continuous predictors. Finally, to evaluate potential sex-related effect modification, interactions between sex and energy-adjusted mineral intake variables were examined by introducing a multiplicative interaction term, sex × mineral density per 1000 kcal, into the regression models. Interaction *p*-values were reported explicitly. It was established a priori that, if the interaction term was statistically significant (*p* < 0.05), analyses would be repeated after stratification by sex. In the absence of statistically significant interaction terms after correction for multiple testing, sex-stratified analyses were interpreted as descriptive and exploratory.

### 2.7. Ethical Considerations

This study was approved on 27 June 2022 by the Drug Research Ethics Committee of the Salamanca Health Area, Spain (CEIm: Ref. PI 2022 06 1048). Throughout the study, the standards of good practice for observational studies established by the Declaration of Helsinki and the World Health Organization were followed [65]. Participant confidentiality was guaranteed in accordance with Spanish Organic Law 3/2018, European Regulation 2016/679, and Council Directive 27 April 2016 on data protection. All participants provided written informed consent before inclusion in the study, after receiving detailed information about the procedures to be performed.

### 2.8. Use of Artificial Intelligence Tools

During manuscript preparation, ChatGPT (OpenAI, GPT-5.5 Thinking) was used to assist with English-language editing and with the formatting and assembly of manuscript figures based on results generated by the authors. Generative artificial intelligence was not used to create scientific images, generate data, perform statistical analyses, or alter the study results. All figures were compared against the original data and statistical outputs by the authors. The authors reviewed and approved all AI-assisted edits and take full responsibility for the final manuscript.

## 3. Results

### 3.1. Study Participants

The characteristics of the study population, both overall and according to sex, are presented in Table 1. Women accounted for 68% of the sample. Men were older than women and had higher alcohol consumption, systolic blood pressure (SBP), diastolic blood pressure (DBP), fasting plasma glucose (FPG), triglyceride levels, and body mass index (BMI). Conversely, high-density lipoprotein cholesterol (HDL-C) levels were higher in women. Regarding vascular assessment, men exhibited higher values for the analyzed vascular structure and function parameters. The mean interval between the diagnosis of acute SARS-CoV-2 infection and inclusion in the study was 38.7 ± 9.6 months.

### 3.2. Dietary Mineral Intake Assessed Using the EVIDENT Tool

Dietary mineral intake estimated from the 7-day dietary record using the EVIDENT tool is presented in Table 2, both for the overall sample and stratified by sex. No statistically significant differences were found between men and women in the intake of the minerals analyzed.

**Figure 2:**
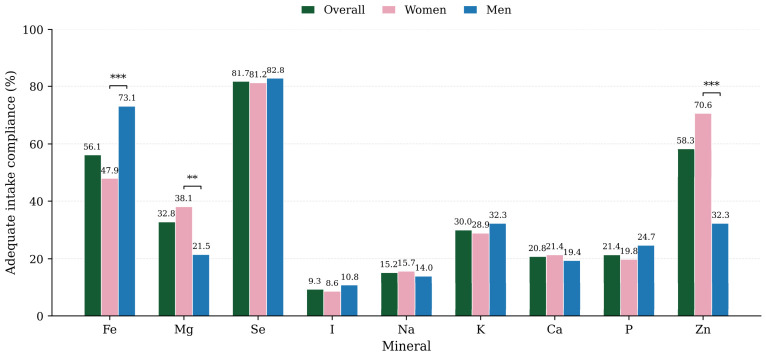
Percentage of participants meeting mineral intake recommendations in the overall sample and by sex. Bars represent the proportion of participants with adequate intake for each mineral. Asterisks indicate statistically significant differences between women and men according to Pearson’s chi-square test: ** *p* < 0.01; *** *p* < 0.001. Abbreviations: Fe, iron; Mg, magnesium; Se, selenium; I, iodine; Na, sodium; K, potassium; Ca, calcium; P, phosphorus; Zn, zinc.

Figure 2 illustrates the proportion of participants meeting adequate mineral intake recommendations in the overall sample and stratified by sex. The proportion of participants achieving adequate intake differed markedly across minerals, being highest for selenium, zinc, and iron, and lowest for iodine, sodium, calcium, and phosphorus. Adequate iron intake was significantly more prevalent among men, whereas women showed significantly higher adherence to magnesium and zinc recommendations. No significant differences between men and women were observed for the other minerals analyzed.

### 3.3. Estimated Marginal Means of Function and Aging Parameters According to Mineral Intake Adequacy

Table 3 shows the multivariable-adjusted estimated marginal means of vascular stiffness and vascular aging parameters according to mineral intake adequacy. Participants with adequate iron intake showed significantly lower baPWV values than those with inadequate or at-risk intake (12.78 [95% CI: 12.46–13.10] vs. 13.35 [95% CI: 12.94–13.76]; *p* = 0.043). For the remaining minerals, no significant differences were found in cfPWV, baPWV, or VAI between participants with adequate and inadequate/risk intake. Although some parameters showed numerically lower values among participants meeting the recommended intake for certain minerals, particularly calcium, magnesium, potassium, and selenium, these differences did not reach statistical significance. Therefore, these estimated marginal mean analyses should be interpreted as descriptive and complementary, and not as primary evidence of mineral-specific associations.

### 3.4. Multiple Linear Regression Analysis of Function, and Aging Parameters in Relation to Mineral Intake Assessed by the EVIDENT Tool

The multicollinearity diagnostics for the fully adjusted sensitivity models are presented in Supplementary Table S3. VIF values were low across all models, with mineral-specific VIFs close to 1 and maximum model VIFs below 2.0, indicating no relevant multicollinearity among the independent variables included in the regression models.

**Figure 3:**
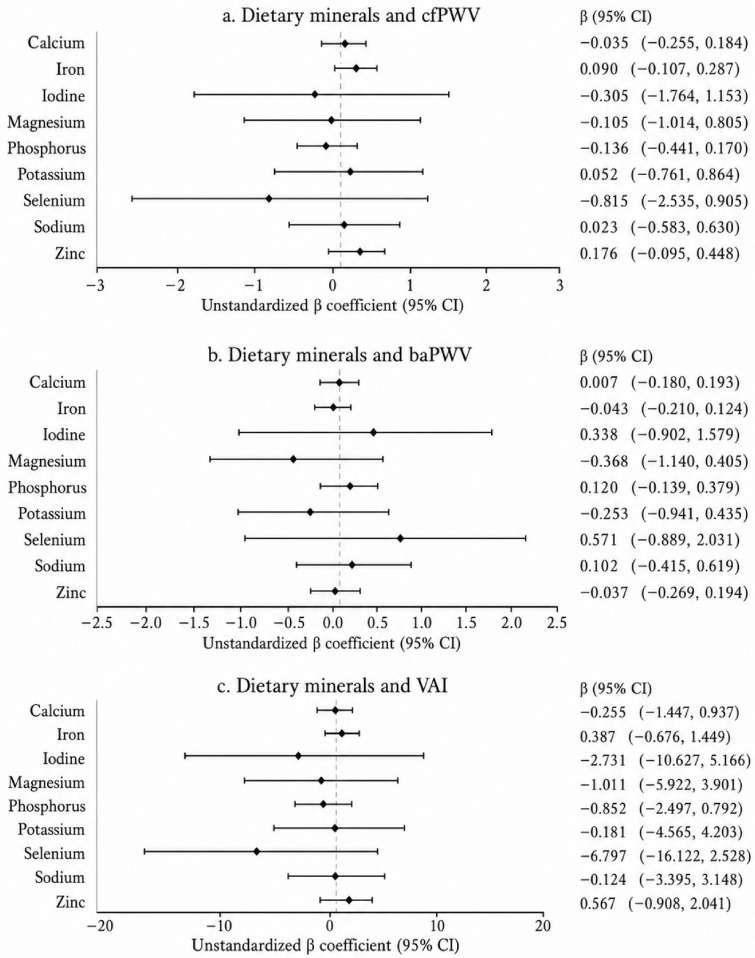
Associations between dietary mineral density and vascular parameters in the total sample (Model 1). Forest plots show the unstandardized β coefficients and 95% confidence intervals from linear regression models evaluating the association of dietary mineral intake density with: (**a**) carotid-femoral pulse wave velocity (cfPWV), (**b**) brachial-ankle pulse wave velocity (baPWV), and (**c**) vascular aging index (VAI). Model 1 was adjusted for age, sex, smoking status, physical activity, and alcohol intake. For graphical display, β coefficients and 95% CIs for calcium, iodine, magnesium, phosphorus, and selenium were multiplied by 100, whereas sodium and potassium were multiplied by 1000. Iron and zinc are shown unscaled.

Figure 3 shows the results of the different energy-adjusted regression models, using mineral intake per 1000 kcal and age, sex, smoking status, physical activity, and alcohol consumption as adjustment variables (Model 1). No significant associations were observed between mineral intake per 1000 kcal and cfPWV, baPWV, or VAI. Although some minerals showed inverse trend estimates, especially selenium, iodine, magnesium and phosphorus for VAI and/or cfPWV.

In Model 1, which included mineral density variables expressed per 1000 kcal and was adjusted for age, sex, smoking status, physical activity, and alcohol intake, no statistically significant linear associations were observed between dietary mineral density and cfPWV, baPWV, or VAI. Although some estimates were directionally inverse, particularly selenium, iodine, magnesium, and phosphorus for VAI and/or cfPWV, all 95% CIs crossed zero and none of the mineral-outcome associations remained significant after FDR correction (Supplementary Table S5).

**Figure 4:**
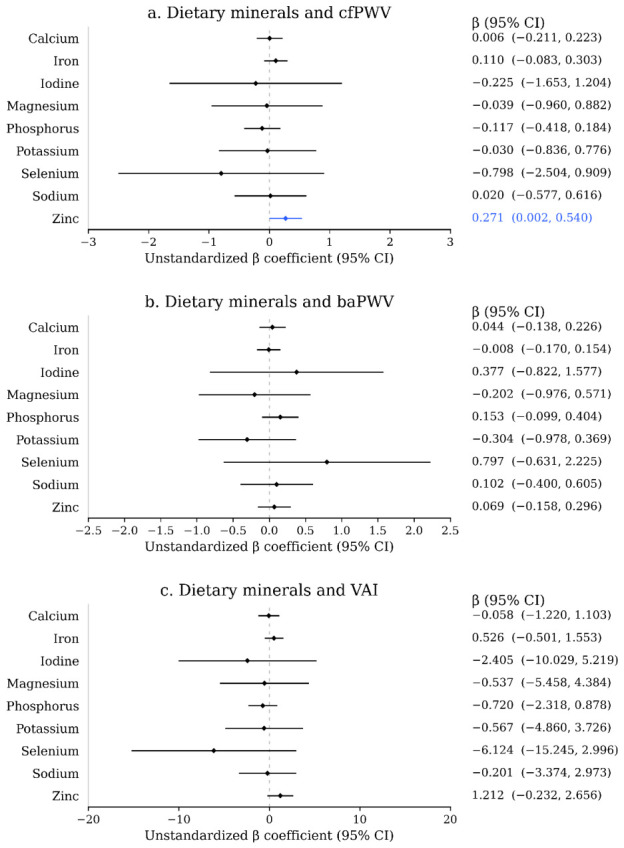
Associations between dietary mineral density and vascular parameters in the total sample (Model 2). Forest plots show the unstandardized β coefficients and 95% confidence intervals from fully adjusted sensitivity linear regression models evaluating the association of dietary mineral intake density with: (**a**) carotid-femoral pulse wave velocity (cfPWV), (**b**) brachial-ankle pulse wave velocity (baPWV), and (**c**) vascular aging index (VAI). Model 2 additionally included Mediterranean diet score, SF-36 score, hypertension, type 2 diabetes mellitus, dyslipidemia, and abdominal obesity. For graphical display, β coefficients and 95% CIs for calcium, iodine, magnesium, phosphorus, and selenium were multiplied by 100, whereas sodium and potassium were multiplied by 1000. Iron and zinc are shown unscaled.

Figure 4 shows the results of the different regression models using fully adjusted Model 2. In this sensitivity model, which included cardiometabolic variables, associations between energy-adjusted mineral intake and vascular parameters were attenuated. Zinc intake per 1000 Kcal was positively associated with cfPWV. No significant associations were observed for the remaining minerals with cfPWV, baPWV, or VAI.

Table S5 shows the regression coefficients between dietary mineral density and the three vascular parameters assessed, along with nominal *p* values and q-values adjusted using the Benjamini–Hochberg procedure. In Model 1, no statistically significant associations were observed between the minerals evaluated and cfPWV, baPWV, or VAI. In Model 2, dietary zinc density showed a nominally significant positive association with cfPWV (B = 0.271; 95% CI: 0.0018 to 0.540; *p* = 0.048). However, this association did not remain significant after FDR correction (q = 0.956). None of the remaining associations between minerals and vascular parameters reached statistical significance after correction for multiple comparisons. Therefore, nominal findings should be interpreted with caution and considered exploratory.

Analyses using restricted cubic splines showed significant associations between dietary density of magnesium and potassium and the vascular parameters assessed (Table S4; Figures S1–S3). For cfPWV, both magnesium and potassium presented significant global associations, with evidence of non-linearity for both minerals. In baPWV, magnesium and potassium were also significantly associated with the outcome, although without evidence of a nonlinear relationship. Similarly, the VAI showed significant and nonlinear global associations with dietary density of magnesium and potassium. Taken together, these results suggest that the associations of magnesium and potassium with cfPWV and VAI might not follow a strictly linear pattern, while their relationship with baPWV appears to be predominantly linear.

As an additional sensitivity analysis, the regression models were repeated after excluding the 8 participants who reported dietary supplement use. In this restricted sample, no mineral-vascular outcome association reached nominal statistical significance or remained significant after FDR correction. The positive association between zinc density and cfPWV observed in the main fully adjusted analysis was attenuated and no longer statistically significant (B = 0.282; 95% CI: −0.017 to 0.580; *p* = 0.064; FDR q = 0.887), suggesting that the main findings were not driven by participants reporting supplement use (Supplementary Table S6).

Finally, formal sex × mineral interaction analyses did not provide robust evidence of sex-related effect modification. Only the selenium × sex interaction for VAI was nominally significant (*p* = 0.035), but it did not remain significant after Benjamini–Hochberg correction across the 27 interaction tests (FDR q = 0.455). Therefore, sex-stratified analyses were interpreted as descriptive and exploratory rather than as evidence of true differences between men and women (Supplementary Table S7 and Supplementary Figure S4).

## 4. Discussion

### 4.1. Main Findings

In this cross-sectional study of 286 adults with LC, the relationship between vascular parameters and mineral intake adjusted per 1000 kcal was analyzed by using hierarchical adjustment and correction models for multiple comparisons. Under this updated analytical strategy, no dietary mineral density-outcome association remained statistically significant after FDR correction. In the fully adjusted sensitivity model, zinc density showed a nominal positive association with cfPWV, but this finding was attenuated after excluding supplement users and did not survive FDR correction. Therefore, the results should be interpreted as exploratory and hypothesis-generating rather than as evidence of robust independent linear effects of individual minerals on vascular stiffness or vascular aging.

Analyses based on mineral intake adequacy categories should be interpreted as complementary sensitivity analyses and not as a direct reproduction of regression models with continuous exposure. While continuous models preserve all the variability of mineral intake, classification into adequate versus inadequate or risk reduces the available information and can decrease statistical power, especially when group sizes are small or unbalanced. Therefore, the differences between continuous models and categorical analyses can be explained by the combined effect of energy adjustment, the loss of information due to dichotomization, and the possible existence of non-linear dose–response relationships.

### 4.2. Absolute Mineral Intake, Arterial Stiffness and Vascular Aging

In this cross-sectional study of adults with LC, we examined the association between energy-adjusted dietary mineral intake and vascular stiffness and vascular aging parameters using hierarchical regression models and correction for multiple comparisons. In addition, restricted cubic spline analyses suggested potential non-linear associations of magnesium and potassium density with cfPWV and VAI. These findings are biologically plausible because magnesium and potassium are involved in vascular tone, endothelial function, nitric oxide bioavailability, electrolyte balance, and smooth-muscle relaxation [24,25,26,27,45,46]. However, because the primary linear regression models did not show FDR-significant associations, these patterns should be interpreted cautiously and require confirmation in studies designed to evaluate dose–response and threshold effects.

The observed distribution of mineral adequacy also deserves attention. Adequacy was higher for selenium, zinc, and iron, whereas lower adherence was observed for calcium, magnesium, potassium, sodium, phosphorus, and iodine. This pattern is consistent with previous studies conducted in Spanish and Mediterranean populations, such as the ANIBES and MEAL studies [59], which reported insufficient intakes of several minerals. Moreover, recent global estimates have identified calcium, iodine, and iron among the micronutrients with the highest prevalence of inadequate intake, with sex-related differences [60]. These findings suggest potential dietary gaps among patients with LC, although they should not be interpreted as biochemical deficiencies in the absence of analytical confirmation.

Phosphorus intake deserves specific consideration because total dietary phosphorus may originate from both naturally occurring organic phosphorus and inorganic phosphate additives [66,67]. Natural sources include dairy products, meat, fish, eggs, legumes, nuts, and cereals [67,68]. In contrast, phosphate additives are frequently used in processed meats, processed cheese, industrial bakery products, ready-to-eat meals, fast foods, cola-type soft drinks, and other ultra-processed foods [66,67,69]. Inorganic phosphate additives are generally more readily absorbable than naturally occurring organic phosphorus and may therefore increase the total phosphorus load disproportionately [66,67,70]. However, the present dietary assessment did not allow us to distinguish phosphorus naturally present in foods from phosphorus derived from additives. Consequently, the interpretation of phosphorus-related findings should be cautious, particularly after energy adjustment and correction for multiple comparisons.

Given the number of comparisons performed, the nominal associations observed in some models should be interpreted with caution. After correction for multiple testing using the Benjamini–Hochberg procedure, none of the 27 primary mineral–vascular outcome associations remained statistically significant. Accordingly, these results should be considered exploratory and hypothesis-generating, particularly for estimates with *p*-values close to 0.05 and for sex-stratified analyses.

Analyses using restricted cubic splines provide additional information by suggesting that associations of dietary density of magnesium and potassium with some vascular parameters may not be strictly linear. In particular, significant nonlinear associations were observed with cfPWV and VAI, while the relationship with baPWV appeared predominantly linear. These findings could indicate the existence of possible thresholds or ranges of intake in which the vascular association of these minerals is more evident. However, given the exploratory nature of these analyses, the number of comparisons made, and the cross-sectional design of the study, the results should be considered hypothesis generators and require confirmation in longitudinal or intervention studies.

Information on dietary supplement use was limited. Only 8 of the 286 participants with available information reported using dietary supplements, corresponding to a prevalence of approximately 2.8%. However, data on supplement type, dose, duration, and mineral composition were not available. Therefore, mineral intake from supplements could not be quantified separately, and we could not determine whether these supplements contained magnesium, calcium, zinc, selenium, or other minerals. Fortified foods may also contribute to mineral intake. In the present study, fortified products were captured only insofar as they were recorded as consumed foods and represented in the food composition database used by the EVIDENT tool. However, minerals derived from fortification could not be separated from naturally occurring minerals, which may have introduced some exposure misclassification.

Sex-stratified analyses were conducted as exploratory analyses and should not be interpreted as evidence of effect modification. Although some associations appeared nominally significant within a single sex stratum, formal sex × mineral interaction tests did not show robust evidence of sex-specific effects after correction for multiple testing. Only the selenium × sex interaction for VAI was nominally significant, but it did not remain significant after Benjamini–Hochberg correction. These findings suggest that the observed sex-stratified patterns may be related to sample size, statistical power, or residual variability rather than true biological differences between men and women.

### 4.3. Clinical Perspective

From a clinical perspective, the present results support the relevance of considering nutritional assessment within the broader follow-up of patients with LC, but they do not justify isolated mineral supplementation or causal claims. Arterial stiffness is a recognized marker of subclinical vascular damage and cardiovascular risk [10,11,12], and LC has increasingly been associated with endothelial dysfunction, persistent inflammation, and accelerated vascular aging [4,8]. In this context, dietary mineral intake and dietary patterns may represent modifiable factors to investigate further. However, future studies should distinguish between mineral intake, food sources, supplement use, fortified foods, additive-derived minerals, and objective biomarkers of mineral status before specific clinical recommendations can be made.

### 4.4. Limitations and Strengths

The main strengths of this study include the relatively large and well-characterized sample of adults with LC, the use of a 7-day dietary record collected with the validated EVIDENT tool, and a comprehensive vascular assessment including cIMT, cfPWV, baPWV, and VAI. In addition, the revised analytical strategy incorporated mineral density variables expressed per 1000 kcal/day, hierarchical regression models, multicollinearity diagnostics, FDR correction for multiple testing, restricted cubic spline analyses, formal sex × mineral interaction testing, and a sensitivity analysis excluding supplement users. Together, these elements strengthen the transparency of the findings and support a cautious interpretation of the results.

Several limitations should be acknowledged. First, the cross-sectional design precludes causal inference and does not exclude reverse causality. Second, dietary intake was self-reported through a 7-day dietary record; although this method provides detailed information, underreporting, portion-size error, recall difficulties, and fatigue-related recording errors in individuals with LC may have affected the accuracy of mineral intake estimates. Third, mineral intake was estimated from dietary records and food composition data, but serum, urinary, or other objective biomarkers of mineral status were not available. Fourth, supplement use was recorded only in a limited way, without information on supplement type, dose, duration, or mineral composition. Fifth, fortified foods and phosphate additives could not be quantified separately, and naturally occurring minerals could not be distinguished from minerals added during processing. Sixth, mineral intakes may partly reflect broader dietary patterns or shared food sources rather than isolated mineral-specific effects. Seventh, residual confounding cannot be excluded, particularly due to unmeasured socioeconomic factors, renal function within the non-excluded range, inflammatory biomarkers, hormonal status, medication use, or detailed food-source information. Finally, although the total sample size was adequate for the overall analyses, the study may have been underpowered to detect small effects, particularly in sex-stratified analyses and interaction testing.

### 4.5. Evidence Gaps and Future Research

Evidence on the relationship between dietary mineral intake and vascular health in LC remains scarce. Future longitudinal studies are needed to determine the temporal direction of the observed associations and to clarify whether mineral intake precedes changes in arterial stiffness or vascular aging. Intervention trials would also be useful to assess whether improving overall diet quality or correcting inadequate mineral intake can modify vascular outcomes in this population. Future research should combine detailed dietary assessment with objective biomarkers of mineral status, including serum, urinary, or functional markers when appropriate. Studies should also quantify the contribution of dietary supplements, fortified foods, and phosphate additives, and should distinguish between naturally occurring minerals and minerals added during food processing. Finally, adequately powered studies are required to evaluate sex-specific patterns using formal interaction testing rather than relying only on stratified analyses.

## 5. Conclusions

After energy adjustment and correction for multiple testing, no robust independent linear association was observed between dietary mineral density and cfPWV, baPWV, or VAI in adults with LC. Descriptive adequacy analyses and restricted cubic spline models suggested possible mineral-related patterns, particularly for iron, magnesium, and potassium, whereas zinc showed only a nominal positive association with cfPWV in the fully adjusted sensitivity model. Formal sex × mineral interaction analyses did not support robust sex-related effect modification. These results highlight the need for prospective studies and nutritional intervention trials integrating dietary sources, supplement use, fortified foods, additive-derived minerals, and objective biomarkers of mineral status to clarify the potential role of mineral intake in vascular health among individuals with LC.

## Figures and Tables

**Table 1:** General characteristics in subjects with LC, overall and by sex.

Variable	Overall (n = 304)	Men (n = 97)	Women (n = 207)	*p*
Age, years (mean ± SD)	52.71 ± 11.94	55.70 ± 12.28	51.32 ± 11.54	0.001
Time of evolution, months (mean ± SD)	38.66 ± 9.58	38.50 ± 9.96	38.74 ± 9.41	0.990
SF-36 HTS, mean ± SD	50.34 ±25.46	51.84 ±25.06	49.63 ±25.67	0.486
Alcohol, g/week (mean ± SD)	29.35 ± 52.87	60.39 ± 76.35	14.88 ± 27.19	<0.001
No alcohol consumption (n, %)	163 (53.6)	36 (37.1)	127 (61.4)	<0.001
MD score (mean ± SD)	7.80 ± 2.33	7.71 ± 2.23	7.84 ± 2.39	0.437
MD adherence (n, %)	123 (40.50)	38 (39.2)	85 (41.1)	0.427
MET-min/week	1226 ± 126	1358 ± 196	1164 ± 75	0.377
Current smoker, (n, %)	17 (5.7)	8 (8.4)	9 (4.5)	0.065
SBP, mmHg (mean ± SD)	119.95 ± 16.75	129.45 ± 14.37	115.52 ± 15.94	<0.001
DBP, mmHg (mean ± SD)	76.85 ± 11.11	82.34 ± 11.04	74.30 ± 10.20	<0.001
Hypertension, (n, %)	109 (35.9)	52 (53.6)	57 (27.5)	<0.001
Total cholesterol, mg/dL (mean ± SD)	187.45 ± 34.30	182.11 ± 32.94	189.95 ± 34.71	0.029
LDL-c, mg/dL (mean ± SD)	113.03 ± 31.76	113.59 ± 32.12	112.77 ± 31.67	0.417
HDL-c, mg/dL (mean ± SD)	56.92 ± 13.58	48.78 ± 10.86	60.73 ± 13.06	<0.001
Triglycerides, mg/dL (mean ± SD)	102.23 ± 50.81	117.47 ± 54.39	95.09 ± 47.52	<0.001
Dyslipidemia, (n, %)	201 (66.3)	71 (73.2)	130 (63.1)	0.053
FPG, mg/dL (mean ± SD)	87.88 ± 17.67	94.37 ± 19.77	84.84 ± 15.74	<0.001
Diabetes mellitus, (n, %)	37 (12.2)	22 (22.7)	15 (7.3)	<0.001
Weight, kg (mean ± SD)	75.95 ± 17.39	88.09 ± 14.95	70.29 ± 15.46	<0.001
Height, cm (mean ± SD)	164.50 ± 8.71	172.51 ± 7.35	160.77 ± 6.52	<0.001
BMI, kg/m^2^ (mean ± SD)	27.97 ± 5.55	29.60 ± 4.64	27.21 ± 5.78	<0.001
Obesity, (n, %)	99 (32.5)	44 (45.4)	55 (26.4)	<0.001
cIMT, mm	0.64 ± 0.09	0.68 ± 0.12	0.62 ± 0.07	<0.001
cfPWV, m/s	7.67 ± 2.36	8.85 ± 2.95	7.12 ± 1.79	<0.001
baPWV, m/s	12.79 ± 2.38	13.63 ± 2.40	12.40 ± 2.27	<0.001
VAI	65.54 ± 13.75	72.89 ± 16.66	62.13 ± 10.59	<0.001

Continuous variables are presented as mean ± standard deviation; between-sex comparisons were performed using Student’s *t*-test. Categorical variables are shown as n (%) and were compared with χ^2^ or Fisher’s exact test, as appropriate. A *p*-value < 0.05 was considered statistically significant. Abbreviations: SD, Standard Deviation; SF-36 HTS, Short Form-36 Health Transition Score; MD, Mediterranean Diet; MET, Metabolic Equivalent; SBP, Systolic Blood Pressure; DBP, Diastolic Blood Pressure; LDL, Low–Density Lipoprotein; HDL, High–Density Lipoprotein; FPG, Fasting Plasma Glucose; BMI, Body Mass Index; cIMT: Intima–Media Thickness of Common Carotid; cfPWV, Carotid-Femoral Pulse Wave Velocity; baPWV, Brachial-Ankle Pulse Wave Velocity; VAI, Vascular Aging Index.

**Table 2:** Mineral and energy intake, overall and by Sex.

Variable	Overall (n = 304)	Men (n = 97)	Women (n = 207)	*p* Value
Energy (kcal/day)	1770 ± 498	1789 ± 529	1762 ± 485	0.666
Fe, mg	13.82 ± 4.03	13.90 ± 4.39	13.78 ± 3.86	0.830
Mg, mg	288.15 ± 82.32	287.78 ± 88.73	288.32 ± 79.35	0.960
Se, µg	99.91 ± 36.76	105.03 ± 39.29	97.50 ± 35.34	0.118
I, µg	98.78 ± 39.29	101.18 ± 41.57	97.65 ± 38.18	0.490
Na, mg	3012 ± 1148	3135 ± 1259	2954 ± 1090	0.235
K, mg	3149 ± 900	3179 ± 950	3135 ± 877	0.705
Ca, mg	767 ± 275	769 ± 267	767 ± 279	0.947
P, mg	429 ± 183	450 ± 204	419 ± 172	0.270
Zn, mg	9.62 ± 2.76	9.65 ± 2.96	9.61 ± 2.67	0.923

Continuous variables are presented as mean ± standard deviation (SD). Fe, iron; Mg, magnesium; Se, selenium; I, iodine; Na, sodium; K, potassium; Ca, calcium; P, phosphorus; Zn, zinc.

**Table 3:** Estimated marginal means of vascular stiffness parameters according to adequate mineral intake.

Mineral	Vascular Parameter	Inadequate/ Risk n	Inadequate/Risk EMM (95% CI)	Adequate n	Adequate EMM (95% CI)	*p* Value
Calcium	cfPWV	228	8.16 (7.85–8.47)	60	7.84 (7.19–8.48)	0.385
Calcium	baPWV	226	13.02 (12.75–13.28)	60	12.91 (12.37–13.46)	0.738
Calcium	VAI	226	68.40 (66.70–70.10)	60	66.71 (63.24–70.19)	0.401
Iron	cfPWV	125	8.32 (7.84–8.81)	159	7.94 (7.56–8.32)	0.247
Iron	baPWV	123	13.35 (12.94–13.76)	159	12.78 (12.46–13.10)	**0.043**
Iron	VAI	124	69.64 (67.03–72.26)	158	67.02 (64.97–69.06)	0.142
Iodine	cfPWV	262	8.08 (7.79–8.37)	27	8.17 (7.28–9.06)	0.845
Iodine	baPWV	260	12.95 (12.71–13.20)	27	13.38 (12.62–14.13)	0.294
Iodine	VAI	260	68.04 (66.47–69.62)	27	67.64 (62.84–72.44)	0.876
Magnesium	cfPWV	194	8.22 (7.87–8.57)	95	7.77 (7.21–8.34)	0.212
Magnesium	baPWV	192	13.05 (12.75–13.34)	95	12.86 (12.38–13.34)	0.540
Magnesium	VAI	193	68.75 (66.87–70.62)	94	66.25 (63.16–69.33)	0.200
Phosphorus	cfPWV	227	8.09 (7.78–8.40)	62	8.06 (7.48–8.65)	0.939
Phosphorus	baPWV	225	12.91 (12.64–13.17)	62	13.28 (12.78–13.78)	0.194
Phosphorus	VAI	226	68.06 (66.36–69.75)	61	67.81 (64.60–71.03)	0.895
Potassium	cfPWV	202	8.05 (7.70–8.40)	87	8.17 (7.61–8.73)	0.736
Potassium	baPWV	200	13.05 (12.76–13.35)	87	12.85 (12.38–13.33)	0.509
Potassium	VAI	201	67.79 (65.89–69.69)	86	68.48 (65.44–71.52)	0.722
Selenium	cfPWV	53	8.31 (7.65–8.97)	236	8.04 (7.73–8.34)	0.467
Selenium	baPWV	53	13.06 (12.50–13.63)	234	12.98 (12.72–13.24)	0.792
Selenium	VAI	52	69.34 (65.75–72.94)	235	67.71 (66.06–69.37)	0.420
Sodium	cfPWV	245	8.05 (7.75–8.35)	44	8.28 (7.55–9.01)	0.577
Sodium	baPWV	244	12.97 (12.72–13.23)	43	13.11 (12.47–13.74)	0.705
Sodium	VAI	244	67.77 (66.13–69.40)	43	69.36 (65.37–73.34)	0.472
Zinc	cfPWV	120	8.09 (7.62–8.55)	169	8.09 (7.65–8.52)	0.995
Zinc	baPWV	120	12.83 (12.44–13.23)	167	13.14 (12.76–13.51)	0.329
Zinc	VAI	118	68.45 (65.93–70.97)	169	67.60 (65.23–69.97)	0.666

Values are estimated marginal means (95% confidence intervals). Models included adequate mineral intake, sex, age, alcohol intake, physical activity, smoking status, and total energy intake; estimated marginal means were evaluated at the mean values of the covariates and adjusted for sex. EMM, estimated marginal mean; CI, confidence interval; cfPWV, carotid–femoral pulse wave velocity; baPWV, brachial–ankle pulse wave velocity; VAI, vascular aging index. *p* values are from Bonferroni-adjusted pairwise comparisons between inadequate/risk and adequate intake. Significant *p* values are shown in bold.

## Data Availability

The data supporting the findings of this study are available on ZENODO at: https://doi.org/10.5281/zenodo.14282873.

## Supplementary Materials

The following supporting information can be downloaded at https://www.mdpi.com/article/10.3390/nu18132140/s1, Table S1: STROBE checklist; Table S2: Cut-off points used to define mineral intake adequacy; Table S3: Multicollinearity diagnostics for the fully adjusted sensitivity models; Table S4: Restricted cubic spline non-linearity analyses; Figures S1–S3: Restricted cubic spline curves for cfPWV, baPWV, and VAI; Table S5: Associations between dietary mineral density and vascular parameters after FDR correction; Table S6: Sensitivity analysis excluding participants reporting dietary supplement use; Table S7: Sex × mineral interaction analyses for vascular outcomes; Figure S4: Sex-stratified associations between dietary mineral density and vascular outcomes.

## Abbreviations

LC: long COVID-19; WHO, World Health Organization; cfPWV, carotid–femoral pulse wave velocity; baPWV, brachial–ankle pulse wave velocity; VAI, vascular aging index; APISAL, Salamanca Primary Care Research Unit; IBSAL, Biomedical Research Institute of Salamanca; REDCap, Research Electronic Data Capture; STROBE, Strengthening the Reporting of Observational Studies in Epidemiology; BMI, body mass index; SBP, systolic blood pressure; DBP, diastolic blood pressure; cIMT, common carotid intima–media thickness; PWV, pulse wave velocity; FPG, fasting plasma glucose; HDL-C, high-density lipoprotein cholesterol; MEDAS, Mediterranean Diet Adherence Screener; SF-36, 36-item Short Form Health Survey; Ca, calcium; P, phosphorus; Mg, magnesium; Fe, iron; Zn, zinc; Na, sodium; K, potassium; I, iodine; Se, selenium; CI, confidence interval; VIF, variance inflation factor.
