# Supplementary material for: Dietary Mineral Intake and Vascular Health in Patients with Long COVID-19: The BioICOPER Study
Source: Nutrients. 2026 Jul 2;18(13):2140. doi: 10.3390/nu18132140 (PMC13363700; doi:10.3390/nu18132140)
Supplement: Supplementary file 1 [file nutrients-18-02140-s001.zip › nutrients-4375252-supplementary.pdf]

Table S1. STROBE Checklist - BioICOPER Study
STROBE Statement—Checklist of items

Items that should be included in reports of cross-sectional studies

Manuscript Title: Dietary Minerals Intake and Vascular Health in Patients with Long COVID. The BioICOPER Study

Target Journal: Nutrients (MDPI)

Cohort Size: n = 304 adults with Long COVID

Core Variables: 9 Minerals (Fe, Mg, Se, Na, K, Ca, P, I, Zinc) | Vascular Markers (cfPWV, baPWV, VAI)

Item No.	Recommendation	Location (Page and Line Numbers)
1 (a)	Indicate the study's design with a commonly used term in the title or the abstract.	Title: Page 1, Lines 2–3<br>Abstract: Page 1, Line 14
1 (b)	Provide in the abstract an informative and balanced summary of what was done and what was found.	Abstract: Page 1, Lines 11–35
2	Explain the scientific background and rationale for the investigation being reported.	Introduction: Pages 2–4, Lines 43–263
3	State specific objectives, including any prespecified hypotheses.	Introduction: Page 4, Lines 254–263
4	Present key elements of study design early in the paper.	Materials and Methods: Page 4, Lines 265–271
5	Describe the setting, locations, and relevant dates, including periods of recruitment, exposure, follow-up, and data collection.	Materials and Methods: Page 4, Lines 266–271
6	Give the eligibility criteria, and the sources and methods of selection of participants.	Materials and Methods: Page 4, Lines 272–283
7	Clearly define all outcomes, exposures, predictors, potential confounders, and effect modifiers. Give diagnostic criteria, if applicable.	Materials and Methods: Page 6, Lines 319–325<br>Materials and Methods: Page 7, Lines 357–363
8	For each variable of interest, give sources of data and details of methods of assessment (measurement). Describe comparability of assessment methods if there is more than one group.	Materials and Methods: Pages 6–7, Sections 2.3 & 2.4
9	Describe any efforts to address potential sources of bias.	Materials and Methods: Page 5, Lines 304–309<br>Materials and Methods: Page 6, Section 2.3
10	Explain how the study size was arrived at.	Materials and Methods: Page 5, Lines 297–302
11	Explain how quantitative variables were handled in the analyses. If applicable, describe which groupings were chosen and why.	Materials and Methods: Page 6, Lines 319–325

Item No.	Recommendation	Location (Page and Line Numbers)

		Materials and Methods: Page 7, Section 2.5
12 (a)	Describe all statistical methods, including those used to control for confounding.	Materials and Methods: Page 7, Lines 346–363
12 (b)	Describe any methods used to examine subgroups and interactions.	Materials and Methods: Pages 7–8, Lines 364–369
12 (c)	Explain how missing data were addressed.	Materials and Methods: Page 5, Figure 1 Flowchart<br>Materials and Methods: Page 7, Lines 314–315
12 (d)	If applicable, describe analytical methods taking account of strategy for sampling.	Materials and Methods:
12 (e)	Describe any sensitivity analyses.	Materials and Methods:
13 (a)	Report numbers of individuals at each stage of study—eg numbers potentially eligible, examined for eligibility, confirmed eligible, included in the study, completing follow-up, and analysed.	Results: Page 5, Figure 1 Flowchart<br>Results: Page 9, Table 1 & Table 2
13 (b)	Give reasons for non-participation at each stage.	Results: Page 5, Figure 1 Flowchart
13 (c)	Consider use of a flow diagram.	Results: Page 5, Figure 1 Flowchart
14 (a)	Give characteristics of study participants (eg demographic, clinical, social) and information on exposures and potential confounders.	Results: Page 9, Table 1 & Table 2
14 (b)	Indicate number of participants with missing data for each variable of interest.	Results: Page 5, Figure 1 Flowchart<br>Results: Page 9, Table 1 & Table 2
15	Report numbers of outcome events or summary measures.	Results: Page 9, Table 1 & Table 2<br>Results: Page 10, Figure 2
16 (a)	Give unadjusted estimates and, if applicable, confounder-adjusted estimates and their precision (eg, 95% confidence interval). Make clear which confounders were adjusted for and why they were included.	Results: Page 9, Lines 333–339<br>Results: Pages 10–11, Table 3
16 (b)	Report category boundaries when continuous variables were categorized.	Materials and Methods: Page 6, Lines 319–325<br>Results: Supplementary Table S2
16 (c)	If relevant, consider translating estimates of relative risk into absolute risk for a meaningful time period.	Not Applicable (N/A)
17	Report other analyses done—eg analyses of subgroups and interactions,	Results: Page 9, Lines
Item No.	Recommendation	Location (Page and Line Numbers)

	and sensitivity analyses.	340–344<br>Results: Supplementary Tables S3 & S4
18	Summarise key results with reference to study objectives.	Discussion: Page 11, Section 4.1
19	Discuss limitations of the study, taking into account sources of potential bias, and imprecision. Discuss both direction and magnitude of any potential bias.	Discussion: Page 14, Limitations Section
20	Give a cautious overall interpretation of results considering objectives, limitations, multiplicity of analyses, results from similar studies, and other relevant evidence.	Discussion: Pages 11–14, Sections 4.1 & 4.2
21	Discuss the generalisability (external validity) of the study results.	Discussion: Page 14, Section 4.2
22	Give the source of funding and the role of the funders for the present study and, if applicable, for the original study on which the present article is based.	Back Matter: Page 15, Funding Section

Table S2. Cut-off points used to define the adequacy of the daily intake of minerals based on the guidelines of the European Food Safety Authority (EFSA) and the Spanish Society of Community Nutrition (SENC).

Mineral	Unity	Cut-off Point (Men)	Cut-Off Point (Women)
Calcium (Ca)	mg/day	1000	1000
Iron (Fe)	mg/ day	11	18
Iodine (I)	µg/ day	150	150
Magnesium (Mg)	mg/ day	350	300
Zinc (Zn)	mg/ day	11	8
Selenio (Se)	µg/ day	70	70
Sodium (Na)	mg/ day	<2000	<2000
Potasio (K)	mg/ day	3500	3500
Phosphorus (P)	mg/ day	550	550

Supplementary Table S3. Multicollinearity diagnostics for the fully adjusted sensitivity models

Outcome	Mineral	Tolerance	VIF mineral	Maximum VIF	Minimum tolerance	Interpretation
cfPWV	Calcium	0.905	1.105	1.607	0.622	No relevant mult.
cfPWV	Iron	0.963	1.039	1.609	0.622	No relevant mult.
cfPWV	Iodine	0.861	1.161	1.656	0.604	No relevant mult.
cfPWV	Magnesium	0.921	1.085	1.608	0.622	No relevant mult.
cfPWV	Phosphorus	0.914	1.094	1.641	0.609	No relevant mult.
cfPWV	Potassium	0.943	1.060	1.635	0.612	No relevant mult.
cfPWV	Selenium	0.840	1.191	1.625	0.615	No relevant mult.
cfPWV	Sodium	0.892	1.121	1.640	0.610	No relevant mult.
cfPWV	Zinc	0.888	1.126	1.626	0.615	No relevant mult.
baPWV	Calcium	0.904	1.106	1.606	0.623	No relevant mult.
baPWV	Iron	0.971	1.030	1.607	0.622	No relevant mult.
baPWV	Iodine	0.853	1.172	1.663	0.601	No relevant mult.
baPWV	Magnesium	0.927	1.079	1.606	0.623	No relevant mult.
baPWV	Phosphorus	0.911	1.098	1.645	0.608	No relevant mult.
baPWV	Potassium	0.906	1.103	1.680	0.595	No relevant mult.
baPWV	Selenium	0.841	1.190	1.642	0.609	No relevant mult.
baPWV	Sodium	0.892	1.122	1.638	0.611	No relevant mult.
baPWV	Zinc	0.892	1.122	1.620	0.617	No relevant mult.
VAI	Calcium	0.905	1.105	1.607	0.622	No relevant mult.
VAI	Iron	0.963	1.039	1.609	0.622	No relevant mult.
VAI	Iodine	0.861	1.161	1.656	0.604	No relevant mult.
VAI	Magnesium	0.977	1.023	1.362	0.734	No relevant mult.
VAI	Phosphorus	0.914	1.094	1.641	0.609	No relevant mult.
VAI	Potassium	0.943	1.060	1.635	0.612	No relevant mult.
VAI	Selenium	0.840	1.191	1.625	0.615	No relevant mult.
VAI	Sodium	0.892	1.121	1.640	0.610	No relevant mult.
VAI	Zinc	0.888	1.126	1.626	0.615	No relevant mult.

Variance inflation factor (VIF) and tolerance values for the mineral exposure variables in the fully adjusted sensitivity models. Values are shown with a maximum of three decimals. Abbreviations: VIF, variance inflation factor; cfPWV, carotid-femoral pulse wave velocity; baPWV, brachial-ankle pulse wave velocity; VAI, vascular aging index, mult, multicollinearity

Table S4. Restricted cubic spline non-linearity analyses for the association between dietary mineral density and vascular outcomes

**Outcome**	**Mineral**	**n**	**p global**	**p no linealidad**
cfPWV	Magnesium	304	0.023	0.028
cfPWV	Potassium	304	0.016	0.006
baPWV	Magnesium	304	0.026	0.779
baPWV	Potassium	304	0.045	0.715
VAI	Magnesium	304	0.015	0.023
VAI	Potassium	304	0.011	0.005

Non-linearity in the association between dietary mineral density and vascular outcomes was assessed using restricted cubic spline models. The global p value indicates the overall significance of the association between each mineral and the corresponding vascular outcome, whereas the p value for non-linearity tests whether the association significantly deviates from linearity. Significant evidence of non-linearity was observed for magnesium and potassium in relation to cfPWV and VAI, but not for baPWV. cfPWV, carotid-femoral pulse wave velocity; baPWV, brachial-ankle pulse wave velocity; VAI, vascular aging index

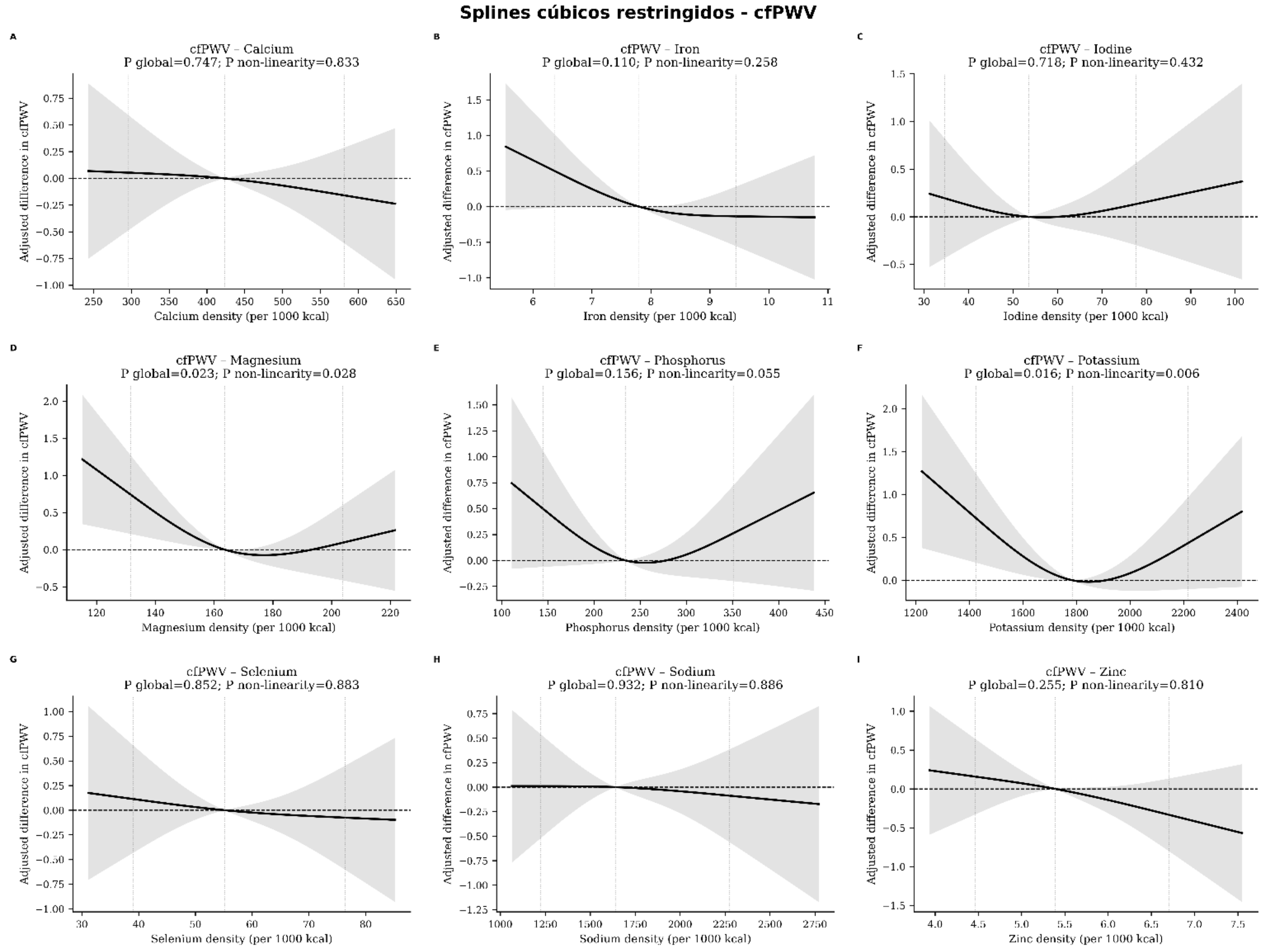

Figure S1. Restricted cubic spline curves showing the adjusted association between dietary mineral density, expressed per 1000 kcal, and carotid–femoral pulse wave velocity (cfPWV). Panels show minerals in alphabetical order: (A) calcium, (B) iron, (C) iodine, (D) magnesium, (E) phosphorus, (F) potassium, (G) selenium, (H) sodium, and (I) zinc. The solid line represents the adjusted difference in cfPWV, and the shaded area represents the 95% confidence interval. Models were adjusted for age, sex, smoking status, alcohol intake, and physical activity. P values for the overall association and non-linearity are shown in each panel.

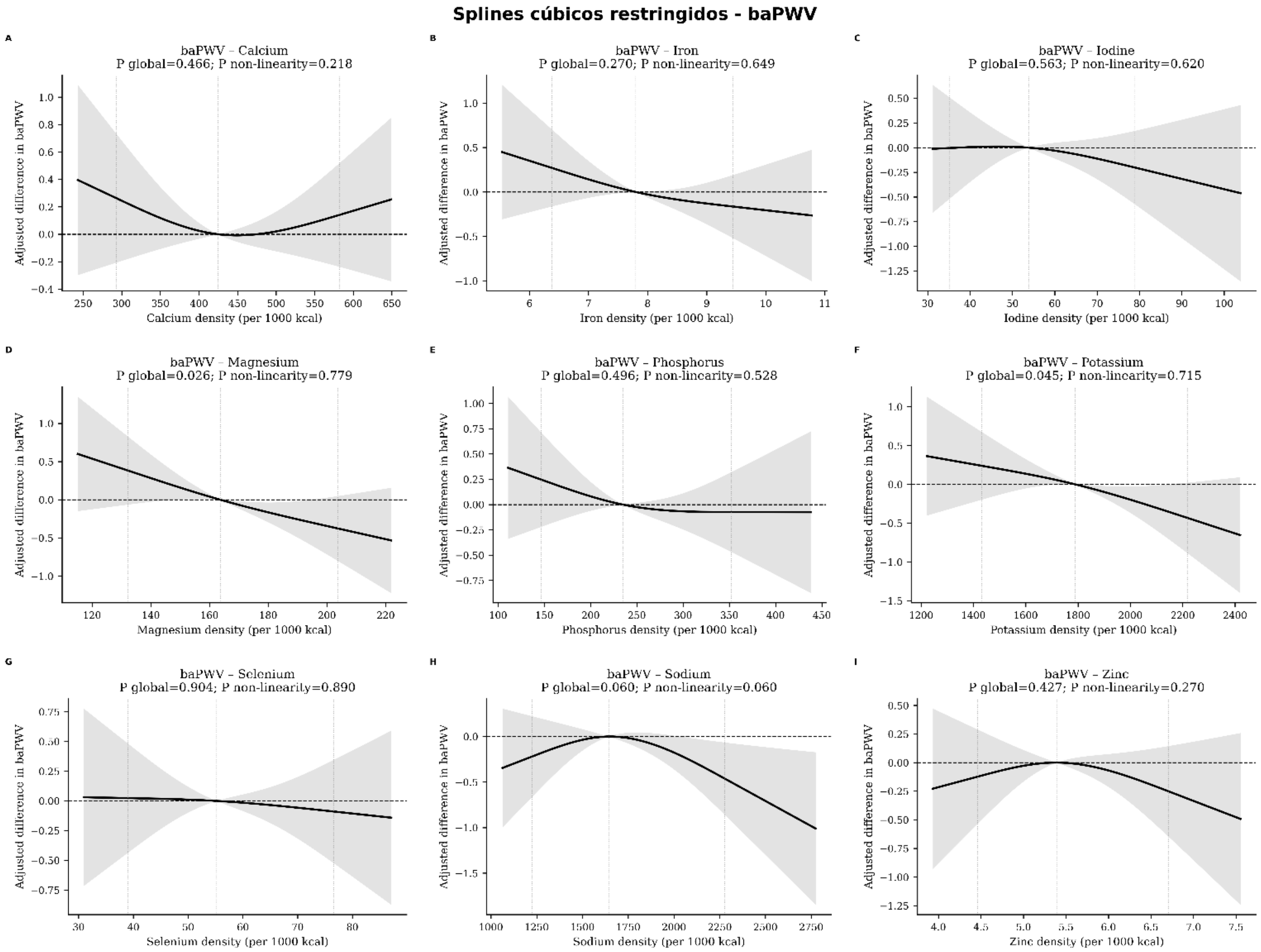

Figure S2. Restricted cubic spline curves showing the adjusted association between dietary mineral density, expressed per 1000 kcal, and brachial–ankle pulse wave velocity (baPWV). Panels show minerals in alphabetical order: (A) calcium, (B) iron, (C) iodine, (D) magnesium, (E) phosphorus, (F) potassium, (G) selenium, (H) sodium, and (I) zinc. The solid line represents the adjusted difference in baPWV, and the shaded area represents the 95% confidence interval. Models were adjusted for age, sex, smoking status, alcohol intake, and physical activity. P values for the overall association and non-linearity are shown in each panel.

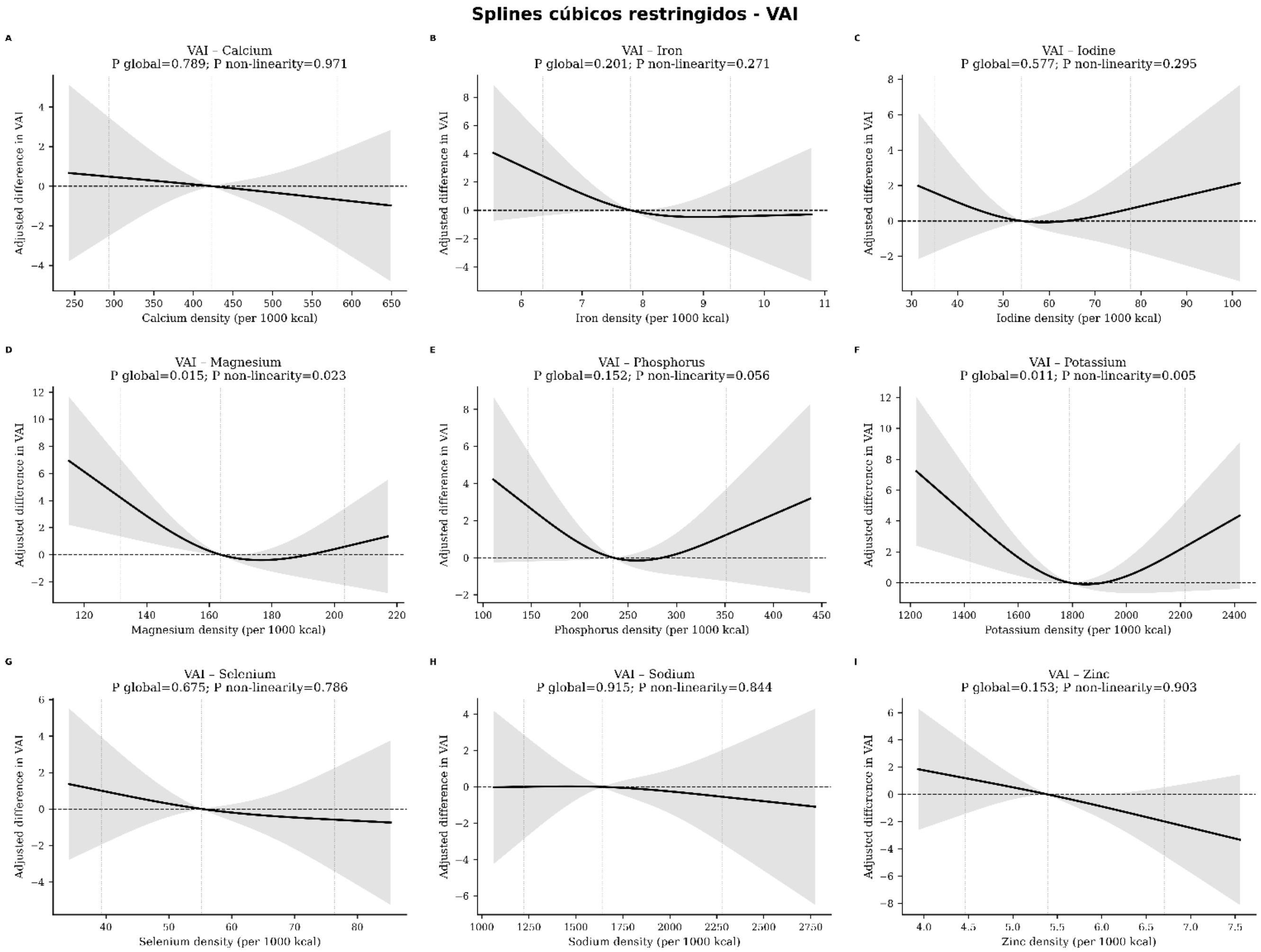

Figure S3. Restricted cubic spline curves showing the adjusted association between dietary mineral density, expressed per 1000 kcal, and vascular aging index (VAI). Panels show minerals in alphabetical order: (A) calcium, (B) iron, (C) iodine, (D) magnesium, (E) phosphorus, (F) potassium, (G) selenium, (H) sodium, and (I) zinc. The solid line represents the adjusted difference in VAI, and the shaded area represents the 95% confidence interval. Models were adjusted for age, sex, smoking status, alcohol intake, and physical activity. P values for the overall association and non-linearity are shown in each panel.

Table S5. Associations between dietary mineral density and vascular parameters after false discovery rate correction

Outcome	Model	Mineral	B (SE)*	95% CI	Unadjusted p-value	FDR q-value
cfPWV	Model 1	Calcium	0.001 (0.001)	-0.003 to 0.002	0.753	0.945
cfPWV	Model 1	Iron	0.090 (0.100)	-0.107 to 0.287	0.370	0.945
cfPWV	Model 1	Iodine	-0.003 (0.007)	-0.018 to 0.012	0.681	0.945
cfPWV	Model 1	Magnesium	-0.001 (0.005)	-0.010 to 0.008	0.821	0.945
cfPWV	Model 1	Phosphorus	-0.001 (0.002)	-0.004 to 0.002	0.382	0.945
cfPWV	Model 1	Potassium	0.001 (0.000)	-0.001 to 0.001	0.900	0.945
cfPWV	Model 1	Selenium	-0.008 (0.009)	-0.025 to 0.009	0.352	0.945
cfPWV	Model 1	Sodium	0.001 (0.000)	-0.001 to 0.001	0.940	0.945
cfPWV	Model 1	Zinc	0.176 (0.138)	-0.095 to 0.448	0.202	0.945
cfPWV	Model 2	Calcium	0.001 (0.001)	-0.002 to 0.002	0.956	0.956
cfPWV	Model 2	Iron	0.110 (0.098)	-0.083 to 0.303	0.263	0.956
cfPWV	Model 2	Iodine	-0.002 (0.007)	-0.017 to 0.012	0.757	0.956
cfPWV	Model 2	Magnesium	0.001 (0.005)	-0.010 to 0.009	0.934	0.956
cfPWV	Model 2	Phosphorus	-0.001 (0.002)	-0.004 to 0.002	0.444	0.956
cfPWV	Model 2	Potassium	0.001 (0.001)	-0.001 to 0.001	0.942	0.956
cfPWV	Model 2	Selenium	-0.008 (0.009)	-0.025 to 0.009	0.358	0.956
cfPWV	Model 2	Sodium	0.001 (0.000)	-0.001 to 0.001	0.949	0.956
cfPWV	Model 2	Zinc	0.271 (0.137)	0.002 to 0.540	0.048	0.956
baPWV	Model 1	Calcium	0.001 (0.001)	-0.002 to 0.002	0.945	0.945
baPWV	Model 1	Iron	-0.043 (0.085)	-0.210 to 0.124	0.611	0.945
baPWV	Model 1	Iodine	0.003 (0.006)	-0.009 to 0.016	0.592	0.945
baPWV	Model 1	Magnesium	-0.004 (0.004)	-0.011 to 0.004	0.350	0.945
baPWV	Model 1	Phosphorus	0.001 (0.001)	-0.001 to 0.004	0.361	0.945
baPWV	Model 1	Potassium	0.001 (0.001)	-0.001 to 0.001	0.469	0.945
baPWV	Model 1	Selenium	0.006 (0.007)	-0.009 to 0.020	0.442	0.945
baPWV	Model 1	Sodium	0.001 (0.001)	0.001 to 0.001	0.699	0.945
baPWV	Model 1	Zinc	-0.037 (0.117)	-0.269 to 0.194	0.751	0.945
baPWV	Model 2	Calcium	0.001 (0.001)	-0.001 to 0.002	0.637	0.956
baPWV	Model 2	Iron	-0.008 (0.082)	-0.170 to 0.154	0.926	0.956
baPWV	Model 2	Iodine	0.004 (0.006)	-0.008 to 0.016	0.536	0.956
baPWV	Model 2	Magnesium	-0.002 (0.004)	-0.010 to 0.006	0.607	0.956
baPWV	Model 2	Phosphorus	0.002 (0.001)	-0.001 to 0.004	0.233	0.956
baPWV	Model 2	Potassium	0.001 (0.001)	-0.001 to 0.001	0.375	0.956
baPWV	Model 2	Selenium	0.008 (0.007)	-0.006 to 0.022	0.273	0.956
baPWV	Model 2	Sodium	0.001 (0.001)	0.001 to 0.001	0.689	0.956
baPWV	Model 2	Zinc	0.069 (0.115)	-0.158 to 0.296	0.549	0.956
VAI	Model 1	Calcium	-0.003 (0.006)	-0.014 to 0.009	0.674	0.945
VAI	Model 1	Iron	0.387 (0.540)	-0.676 to 1.449	0.474	0.945
VAI	Model 1	Iodine	-0.027 (0.040)	-0.106 to 0.052	0.497	0.945
VAI	Model 1	Magnesium	-0.010 (0.025)	-0.059 to 0.039	0.686	0.945
VAI	Model 1	Phosphorus	-0.009 (0.008)	-0.025 to 0.008	0.308	0.945
VAI	Model 1	Potassium	0.001 (0.002)	-0.005 to 0.004	0.935	0.945
VAI	Model 1	Selenium	-0.068 (0.047)	-0.161 to 0.025	0.152	0.945

Outcome	Model	Mineral	B (SE)*	95% CI	Unadjusted p-value	FDR q-value
VAI	Model 1	Sodium	0.001 (0.002)	-0.003 to 0.003	0.941	0.945
VAI	Model 1	Zinc	0.567 (0.749)	-0.908 to 2.041	0.450	0.945
VAI	Model 2	Calcium	-0.001 (0.006)	-0.012 to 0.011	0.921	0.956
VAI	Model 2	Iron	0.526 (0.522)	-0.501 to 1.553	0.314	0.956
VAI	Model 2	Iodine	-0.024 (0.039)	-0.100 to 0.052	0.535	0.956
VAI	Model 2	Magnesium	-0.005 (0.025)	-0.055 to 0.044	0.830	0.956
VAI	Model 2	Phosphorus	-0.007 (0.008)	-0.023 to 0.009	0.376	0.956
VAI	Model 2	Potassium	-0.001 (0.002)	-0.005 to 0.004	0.795	0.956
VAI	Model 2	Selenium	-0.061 (0.046)	-0.152 to 0.030	0.187	0.956
VAI	Model 2	Sodium	0.001 (0.002)	-0.003 to 0.003	0.901	0.956
VAI	Model 2	Zinc	1.212 (0.733)	-0.232 to 2.656	0.100	0.956

Values are expressed as regression coefficient (standard error). Dietary mineral intake was expressed as nutrient density per 1000 kcal/day. Model 1 was adjusted for age, sex, smoking status, alcohol intake, and physical activity. Model 2 was additionally adjusted for hypertension, dyslipidemia, diabetes, obesity, MEDAS and SF-36. Unadjusted p-values correspond to the original regression models. FDR q-values were calculated using the Benjamini–Hochberg procedure to account for multiple comparisons. Abbreviations: baPWV, brachial–ankle pulse wave velocity; B, regression coefficient; cfPWV, carotid–femoral pulse wave velocity; CI, confidence interval; FDR, false discovery rate; SE, standard error; VAI, vascular aging index.

Supplementary Table S6. Sensitivity analysis excluding participants reporting dietary supplement use

Outcome	Mineral	B (SE)	95% CI	Unadjusted p-value	FDR q-value
cfPWV	Calcium	-0.001 (0.001)	-0.003 to 0.002	0.601	0.887
cfPWV	Iron	0.159 (0.114)	-0.066 to 0.384	0.166	0.887
cfPWV	Iodine	-0.003 (0.008)	-0.019 to 0.014	0.745	0.887
cfPWV	Magnesium	-0.002 (0.005)	-0.013 to 0.008	0.661	0.887
cfPWV	Phosphorus	-0.001 (0.002)	-0.005 to 0.002	0.486	0.887
cfPWV	Potassium	0.001(0.000)	-0.001 to 0.001	0.955	0.955
cfPWV	Selenium	-0.005 (0.010)	-0.024 to 0.015	0.645	0.887
cfPWV	Sodium	0.001(0.000)	-0.001 to 0.001	0.664	0.887
cfPWV	Zinc	0.282 (0.152)	-0.017 to 0.580	0.064	0.887
baPWV	Calcium	0.001(0.001)	-0.002 to 0.002	0.757	0.887
baPWV	Iron	0.008 (0.101)	-0.191 to 0.206	0.941	0.955
baPWV	Iodine	0.003 (0.007)	-0.012 to 0.018	0.683	0.887
baPWV	Magnesium	-0.003 (0.005)	-0.012 to 0.007	0.571	0.887
baPWV	Phosphorus	0.001 (0.002)	-0.002 to 0.004	0.423	0.887
baPWV	Potassium	0.001(0.001)	-0.001 to 0.000	0.427	0.887
baPWV	Selenium	0.007 (0.009)	-0.010 to 0.024	0.445	0.887
baPWV	Sodium	0.001(0.001)	0.001to 0.001	0.691	0.887
baPWV	Zinc	-0.030 (0.134)	-0.294 to 0.234	0.822	0.887
VAI	Calcium	-0.005 (0.007)	-0.017 to 0.008	0.478	0.887
VAI	Iron	0.737 (0.611)	-0.467 to 1.940	0.229	0.887
VAI	Iodine	-0.029 (0.045)	-0.118 to 0.060	0.526	0.887
VAI	Magnesium	-0.022 (0.028)	-0.078 to 0.034	0.435	0.887
VAI	Phosphorus	-0.007 (0.009)	-0.025 to 0.011	0.448	0.887
VAI	Potassium	-0.001 (0.003)	-0.006 to 0.004	0.820	0.887
VAI	Selenium	-0.043 (0.053)	-0.148 to 0.061	0.414	0.887
VAI	Sodium	0.001 (0.002)	-0.003 to 0.004	0.727	0.887
VAI	Zinc	1.050 (0.817)	-0.559 to 2.660	0.199	0.887

Values are unstandardized regression coefficients (B), standard errors (SE), 95% confidence intervals (CI), nominal p values, and Benjamini-Hochberg false discovery rate (FDR) q values. The table includes only participants classified as non-users of dietary supplements in the SPSS split-file output. Dietary mineral density was expressed per 1000 kcal. Models were adjusted for age, sex, alcohol intake, physical activity (T_METS_min), and smoking status. FDR correction was applied across the 27 mineral-outcome associations in this sensitivity analysis. Abbreviations: cfPWV, carotid-femoral pulse wave velocity; baPWV, brachial-ankle pulse wave velocity; VAI, vascular aging index; CI, confidence interval; FDR, false discovery rate.

Supplementary Table S7. Sex × mineral interaction analyses for vascular outcomes.

Outcome	Mineral	Sex × mineral interaction B (SE)	95% CI	p for interaction	FDR q-value	Interpretation
cfPWV	Calcium	0.001 (0.003)	-0.004 to 0.006	0.771	0.856	No significant
cfPWV	Iron	-0.218 (0.213)	-0.638 to 0.202	0.308	0.639	No significant
cfPWV	Iodine	0.025 (0.015)	-0.006 to 0.055	0.113	0.455	No significant
cfPWV	Magnesium	0.002 (0.010)	-0.017 to 0.021	0.823	0.856	No significant
cfPWV	Phosphorus	0.006 (0.004)	-0.001 to 0.013	0.088	0.455	No significant
cfPWV	Potassium	0.001(0.001)	-0.002 to 0.002	0.822	0.856	No significant
cfPWV	Selenium	0.028 (0.018)	-0.007 to 0.063	0.118	0.455	No significant
cfPWV	Sodium	0.001 (0.001)	-0.001 to 0.002	0.371	0.639	No significant
cfPWV	Zinc	-0.538 (0.308)	-1.140 to 0.068	0.081	0.455	No significant
baPWV	Calcium	0.001 (0.002)	-0.003 to 0.006	0.541	0.856	No significant
baPWV	Iron	0.040 (0.181)	-0.315 to 0.396	0.824	0.856	No significant
baPWV	Iodine	0.006 (0.013)	-0.019 to 0.032	0.626	0.856	No significant
baPWV	Magnesium	0.009 (0.008)	-0.007 to 0.025	0.274	0.639	No significant
baPWV	Phosphorus	0.003 (0.003)	-0.003 to 0.009	0.351	0.639	No significant
baPWV	Potassium	0.001(0.001)	-0.001 to 0.002	0.856	0.856	No significant
baPWV	Selenium	0.020 (0.015)	-0.010 to 0.050	0.182	0.615	No significant
baPWV	Sodium	0.001(0.001)	-0.001 to 0.001	0.789	0.856	No significant
baPWV	Zinc	-0.244 (0.263)	-0.761 to 0.274	0.354	0.639	No significant
VAI	Calcium	0.005 (0.014)	-0.023 to 0.034	0.722	0.856	No significant
VAI	Iron	-1.02 (1.15)	-3.28 to 1.25	0.378	0.639	No significant
VAI	Iodine	0.156 (0.084)	-0.0086 to 0.320	0.063	0.455	No significant
VAI	Magnesium	0.019 (0.052)	-0.084 to 0.123	0.714	0.856	No significant
VAI	Phosphorus	0.036 (0.019)	-0.0012 to 0.073	0.058	0.455	No significant
VAI	Potassium	0.0021 (0.0048)	-0.0073 to 0.012	0.658	0.856	No significant
VAI	Selenium	0.204 (0.096)	0.014 to 0.394	0.035	0.455	No significant
VAI	Sodium	0.0039 (0.0036)	-0.0032 to 0.011	0.276	0.639	No significant
VAI	Zinc	-1.85 (1.69)	-5.16 to 1.47	0.275	0.639	No significant

Values correspond to the formal sex × mineral interaction terms from the regression models including mineral density, sex, and the sex × mineral interaction term, adjusted for age, alcohol intake, physical activity, and smoking status. Dietary mineral density was expressed per 1000 kcal. FDR q-values were calculated using the Benjamini–Hochberg procedure across the 27 interaction tests. A nominal interaction was observed only for selenium × sex in relation to VAI; however, this did not remain significant after FDR correction. Therefore, sex-stratified analyses should be interpreted as descriptive and exploratory rather than as evidence of robust effect modification by sex. Abbreviations: B, unstandardized beta coefficient; CI, confidence interval; cfPWV, carotid–femoral pulse wave velocity; baPWV, brachial–ankle pulse wave velocity; VAI, vascular aging index; FDR, false discovery rate.

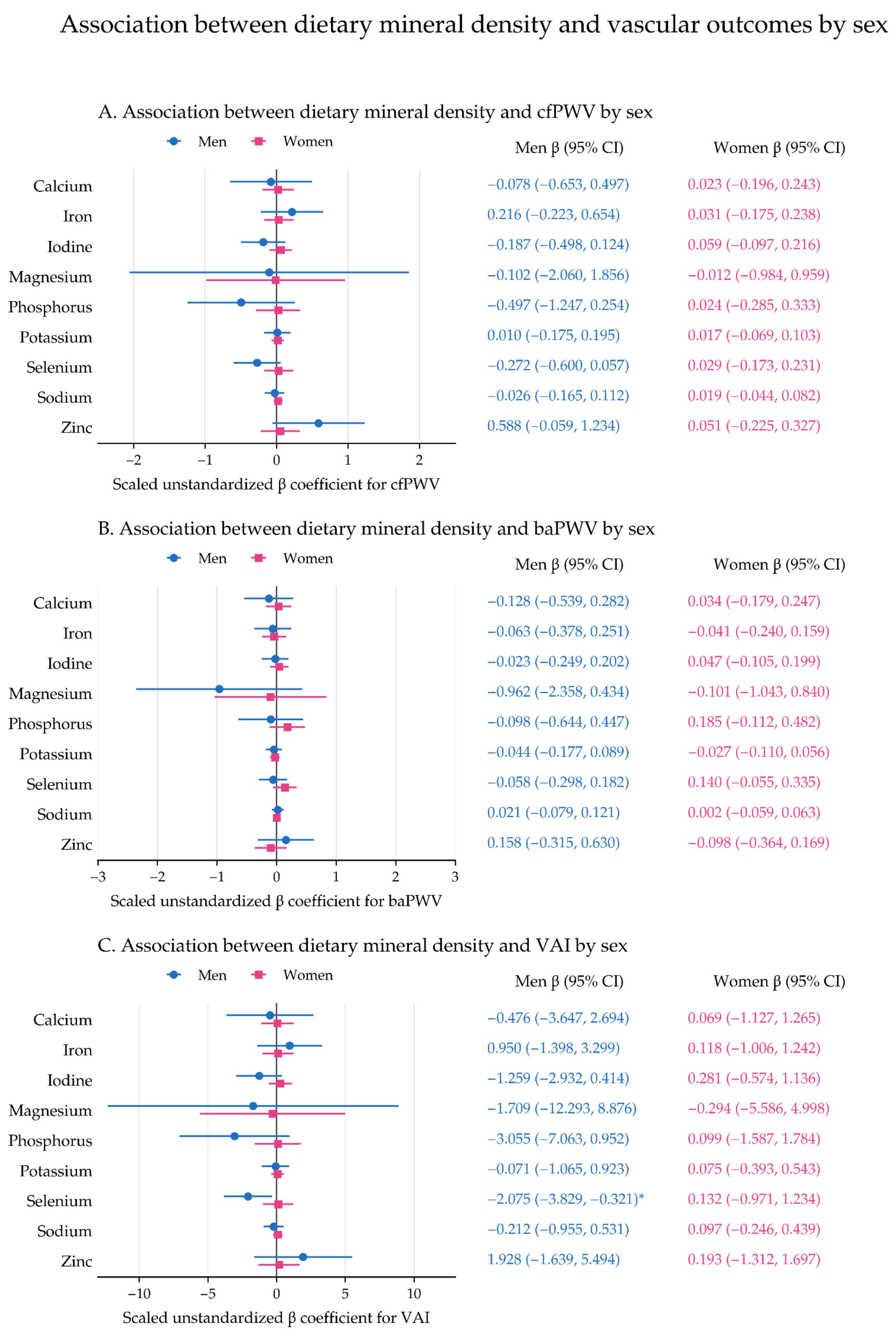
Association between dietary mineral density and vascular outcomes by sex
A. Association between dietary mineral density and cfPWV by sex
Men
Women
Men β (95% CI)
Women β (95% CI)
Calcium
Iron
Iodine
Magnesium
Phosphorus
Potassium
Selenium
Sodium
Zinc
−0.078 (−0.653, 0.497)
0.023 (−0.196, 0.243)
0.216 (−0.223, 0.654)
0.031 (−0.175, 0.238)
−0.187 (−0.498, 0.124)
0.059 (−0.097, 0.216)
−0.102 (−2.060, 1.856)
−0.012 (−0.984, 0.959)
−0.497 (−1.247, 0.254)
0.024 (−0.285, 0.333)
0.010 (−0.175, 0.195)
0.017 (−0.069, 0.103)
−0.272 (−0.600, 0.057)
0.029 (−0.173, 0.231)
−0.026 (−0.165, 0.112)
0.019 (−0.044, 0.082)
0.588 (−0.059, 1.234)
0.051 (−0.225, 0.327)
−2
−1
0
1
2
Scaled unstandardized β coefficient for cfPWV
B. Association between dietary mineral density and baPWV by sex
Men
Women
Men β (95% CI)
Women β (95% CI)
Calcium
Iron
Iodine
Magnesium
Phosphorus
Potassium
Selenium
Sodium
Zinc
−0.128 (−0.539, 0.282)
0.034 (−0.179, 0.247)
−0.063 (−0.378, 0.251)
−0.041 (−0.240, 0.159)
−0.023 (−0.249, 0.202)
0.047 (−0.105, 0.199)
−0.962 (−2.358, 0.434)
−0.101 (−1.043, 0.840)
−0.098 (−0.644, 0.447)
0.185 (−0.112, 0.482)
−0.044 (−0.177, 0.089)
−0.027 (−0.110, 0.056)
−0.058 (−0.298, 0.182)
0.140 (−0.055, 0.335)
0.021 (−0.079, 0.121)
0.002 (−0.059, 0.063)
0.158 (−0.315, 0.630)
−0.098 (−0.364, 0.169)
−3
−2
−1
0
1
2
3
Scaled unstandardized β coefficient for baPWV
C. Association between dietary mineral density and VAI by sex
Men
Women
Men β (95% CI)
Women β (95% CI)
Calcium
Iron
Iodine
Magnesium
Phosphorus
Potassium
Selenium
Sodium
Zinc
−0.476 (−3.647, 2.694)
0.069 (−1.127, 1.265)
0.950 (−1.398, 3.299)
0.118 (−1.006, 1.242)
−1.259 (−2.932, 0.414)
0.281 (−0.574, 1.136)
−1.709 (−12.293, 8.876)
−0.294 (−5.586, 4.998)
−3.055 (−7.063, 0.952)
0.099 (−1.587, 1.784)
−0.071 (−1.065, 0.923)
0.075 (−0.393, 0.543)
−2.075 (−3.829, −0.321)*
0.132 (−0.971, 1.234)
−0.212 (−0.955, 0.531)
0.097 (−0.246, 0.439)
1.928 (−1.639, 5.494)
0.193 (−1.312, 1.697)
−10
−5
0
5
10
Scaled unstandardized β coefficient for VAI

Figure S4. Sex-stratified associations between dietary mineral density and vascular outcomes. Panels show scaled unstandardized β coefficients and 95% confidence intervals for the associations between dietary mineral density and cfPWV (A), baPWV (B), and VAI (C), separately in men and women. Points represent β coefficients and horizontal lines represent 95% CIs. Numerical β values and 95% CIs are shown on the right side of each panel. Coefficients were scaled for graphical presentation. Models were adjusted as described in the Methods section. $p < 0.05$. cfPWV, carotid-femoral pulse wave velocity; baPWV, brachial-ankle pulse wave velocity; VAI, visceral adiposity index; CI, confidence interval. Blue markers represent men and red markers represent women.
